# The immunotherapy candidate TNFSF4 may help the induction of a promising immunological response in breast carcinomas

**DOI:** 10.1038/s41598-021-98131-4

**Published:** 2021-09-20

**Authors:** Kai Li, Lei Ma, Ye Sun, Xiang Li, Hong Ren, Shou-Ching Tang, Xin Sun

**Affiliations:** 1 Department of Thoracic Surgery, The First Affiliated Hospital of Xi’an Jiaotong University, 277 Yanta West Road, Xi’an, 710061 Shaanxi China; 2 Department of Thoracic Surgery and Oncology, Cancer Centre, The First Affiliated Hospital of Xi’an Jiaotong University, 277 Yanta West Road, Xi’an, 710061 Shaanxi China; 3 Department of Anesthesiology and Perioperative Medicine, Operating Centre, The First Affiliated Hospital of Xi’an Jiaotong University, Xi’an, 710061 Shaanxi China; 4 Department of Anesthesiology and Operation, Operating Centre, The First Affiliated Hospital of Xi’an Jiaotong University, Xi’an, 710061 Shaanxi China; 5 Department of Pathology, Anatomy and Cell Biology, Sidney Kimmel Cancer Center, Thomas Jefferson University, Philadelphia, PA 19107 USA; 6 University of Mississippi Medical Center, Cancer Center and Research Institute, University of Mississippi, 2500 North State Street, Jackson, MS 39216 USA

**Keywords:** Cancer, Cell biology, Computational biology and bioinformatics, Genetics, Immunology, Molecular biology, Stem cells, Biomarkers, Molecular medicine, Oncology, Risk factors

## Abstract

Immune checkpoint blockade, an immunotherapy, has been applied in multiple systemic malignancies and has improved overall survival to a relatively great extent; whether it can be applied in breast cancer remains unknown. We endeavored to explore possible factors that may influence immunotherapy outcomes in breast cancer using several public databases. The possible treatment target TNF superfamily member 4 (TNFSF4) was selected from many candidates based on its abnormal expression profile, survival-associated status, and ability to predict immune system reactions. For the first time, we identified the oncogenic features of TNFSF4 in breast carcinoma. TNFSF4 was revealed to be closely related to treatment that induced antitumor immunity and to interact with multiple immune effector molecules and T cell signatures, which was independent of endocrine status and has not been reported previously. Moreover, the potential immunotherapeutic approach of TNFSF4 blockade showed underlying effects on stem cell expansion, which more strongly and specifically demonstrated the potential effects of applying TNFSF4 blockade-based immunotherapies in breast carcinomas. We identified potential targets that may contribute to breast cancer therapies through clinical analysis and real-world review and provided one potential but crucial tool for treating breast carcinoma that showed effects across subtypes and long-term effectiveness.

## Introduction

Breast carcinoma treatments have been evolving for years, accompanied by the emergence of reagents referred to as endocrine therapy and targeted therapy and improvements in chemotherapeutics^1,2^. However, the development of therapies seems to have encountered a bottleneck, and what approach should be pursued to further prolong patient survival is unclear^3–5^. Can we draw lessons from the experience of immunotherapy?

Improving cancer-related immune resistance is mainly achieved through immune checkpoint blockade therapy, and multiple immune checkpoint-targeting agents have been identified and clinically applied to treat lung cancer, esophageal cancer, melanoma, etc. Therapeutic targets include but are not limited to PD-L1 (CD274), PD-L2 (PDCD1LG2), CD80, CD86, and CD70, and novel applied therapies have shown promise in lung cancer and melanoma treatment^6–10^. However, how immune checkpoint blockade therapy acts in the breast cancer treatment process and what checkpoint may be targeted to achieve benefits are totally unknown. Recently, CCR9 was demonstrated to exert strong immunoregulatory effects on T cell responses in multiple tumors, and through inhibiting TCR signaling, CCR9 regulates STAT signaling in T cells, resulting in reduced T helper 1 cytokine secretion. Unlike PD-L1 blockade, inhibition of CCR9 expression on tumor cells facilitates immunotherapeutic effects via tumor-specific T cells in vivo^11,12^. However, whether and how such immune checkpoints are involved in breast cancer prognosis and therapeutic efficacy remain largely unclear.

Therefore, this study aimed to clarify the clinical significance of immunotherapies, especially the uncertain efficacy of immune checkpoint blockade, in breast cancer patients and to propose more suitable strategies for improving breast cancer treatments by applying our knowledge on immunity.

## Results

### Screening possible immune functions related to anticancer treatment

Common immune checkpoints were browsed and screened for possible immunotherapeutic value, and the representative immune checkpoint molecules^13–15^ ADORA2A, BTLA, Nectin-2 (CD112), CD160, CD244, PD-L1, CD96, CSF1R, CTLA4, HAVCR2, IDO1, IL10, IL10RB, KDR, KIR2DL1, KIR2DL3, LAG3, LGALS9, PDCD1, PDCD1LG2, PVRL2, TGFB1, TGFBR1, TIGIT, VTCN1, TNF Receptor Superfamily Member 14 (TNFRSF14), TNF superfamily member 4 (TNFSF4), and TNF superfamily member 18 (TNFSF18) were all input into the Tumor and Immune System Interaction Database (TISIDB) for potential effect predication in the integrated repository portal for tumor-immune system interactions^16^. Primarily, PD-L1, CD112, TNFRSF14, TNFSF4, TNFSF18, CD48, and LGALS9 were selected for their significant differential expression patterns, and the patterns are shown in Fig. S1. The paired red and green colors indicate the expression patterns in multiple organs and systems.

**Figure 1:**
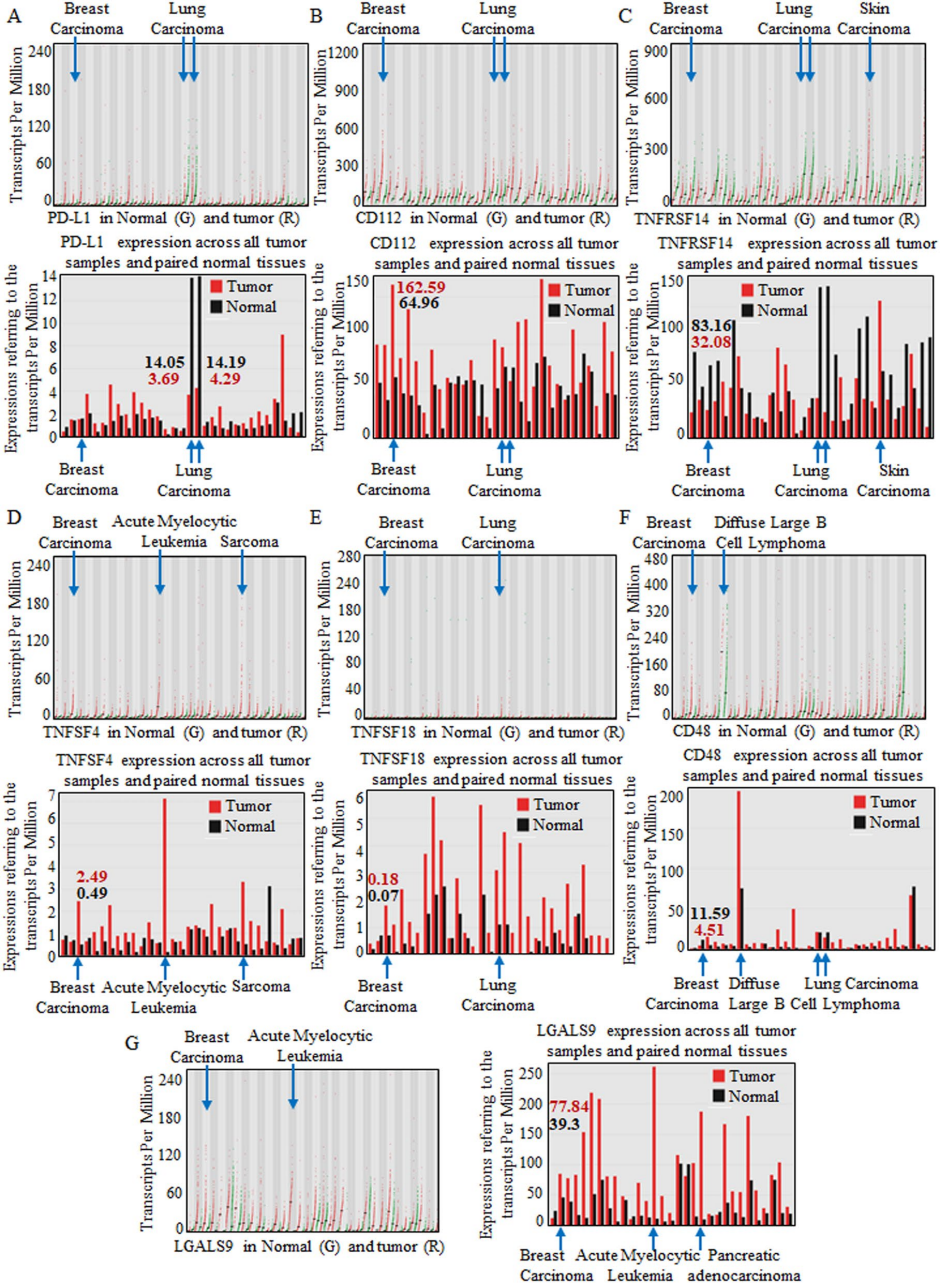
Analyzing candidate immune checkpoint molecules targeted by blockade in carcinomas. Carcinomas throughout the body were enrolled for analysis, and multiple systems showed various immune checkpoint molecule patterns. PD-L1 (**A**), CD112 (**B**), TNFRSF14 (**C**), TNFSF4 (**D**), TNFSF18 (**E**), CD48 (**F**), and LGALS9 (**G**) were analyzed for abnormal expression in breast carcinomas. In each image, the specific quantitative value is shown at the top, and each dot represents the expression in samples. The bar graphs below were used for comparison, and bar height bar represents the median expression of the specific tumor type or normal tissue. Specifically, CD112, TNFSF4, TNFSF18, and LGALS9 were relatively overexpressed in breast carcinomas.

Furthermore, PD-L1 (Fig. 1A), CD112 (Fig. 1B), TNFRSF14 (Fig. 1C), TNFSF4 (Fig. 1D), TNFSF18 (Fig. 1E), CD48 (Fig. 1F), and LGALS9 (Fig. 1G) were analyzed for abnormal expression patterns in breast carcinomas. Specifically, CD112, TNFSF4, TNFSF18, and LGALS9 were significantly overexpressed in breast carcinomas, strongly suggesting potent and valuable effects.

### Immunotherapeutic targets participated in multiple breast carcinogenic processes by interacting with key cancer-stimulating factors

**Figure 2:**
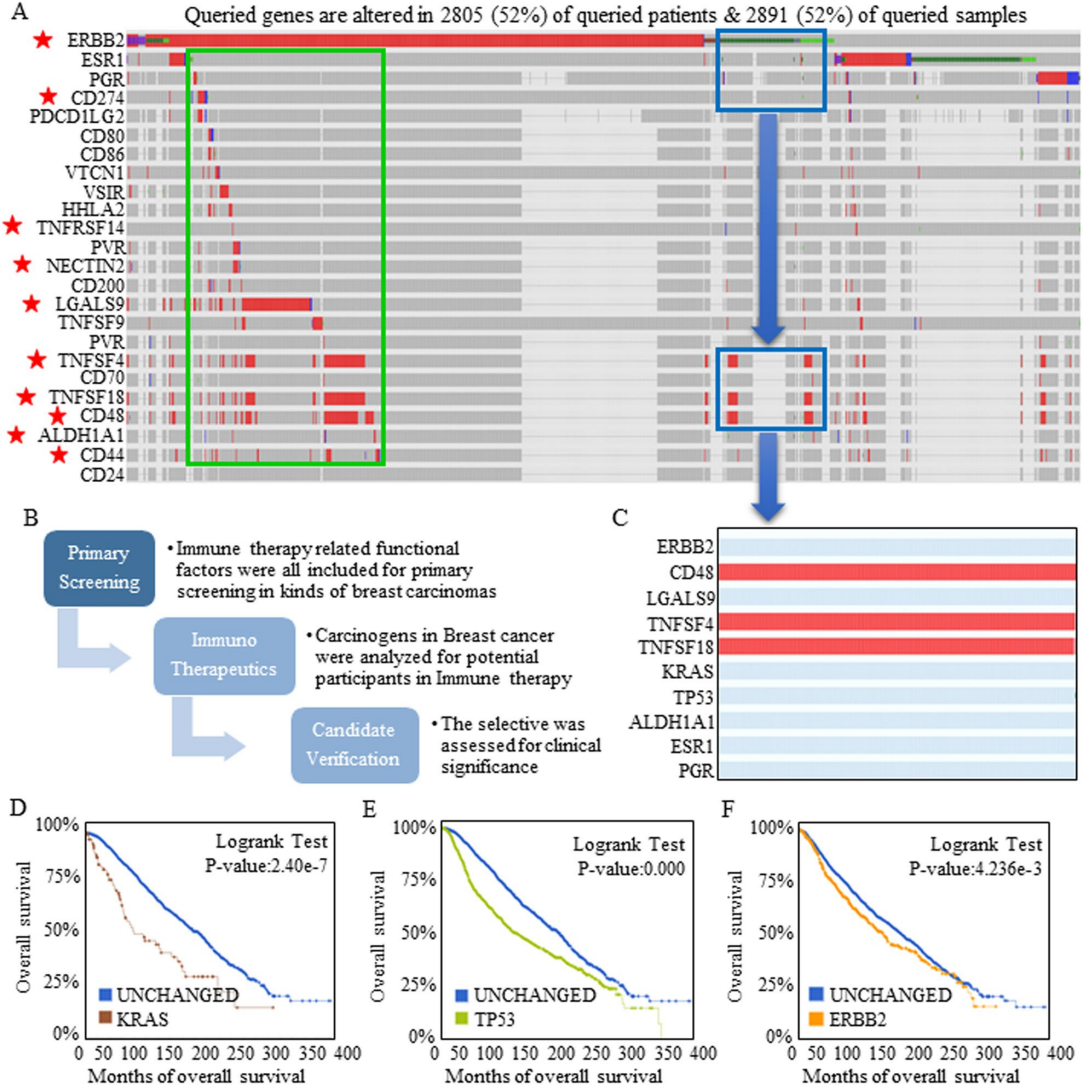
Selection of the most representative immune checkpoint molecules targeted by blockade. Expression signatures and correlations were studied using cBioPortal and GEPIA2. (**A**) Expression heat-map showing the expression of each candidate in a red bar, and the most representative genes are labeled with a red star. The red bars indicate actual data points, and the gray bars indicate that no specific data were available. (**B**) The analysis flowchart is shown. (**C**) Nearly all the immunotherapeutic targets showed expanded expression intervals referring to ESR1 and PGR, and TNFSF4, TNFSF18, and CD48 showed increased expression in breast carcinomas without ERBB2, ESR1, and PGR expression. Amplification or mutation of KRAS (**D**), TP53 (**E**), or ERBB2 (**F**) indicated worse predicted survival.

The heterogenetic features of breast cancer cause breast carcinomas with different hormone receptor statuses to benefit from different and specific treatment strategies. As a double-edged sword, a specific agent will not function in another kind of breast carcinoma, and to determine the potential of immune checkpoint blockade therapies, native expression signatures and correlations were studied using cBioPortal and Gene Expression Profiling Interactive Analysis 2 (GEPIA2). An expression heat-map was used to visualize the expression patterns of the candidates in red bars, and the selected representative genes described in the first part of Result section are labeled with a red star (Fig. 2A). The analysis flowchart is shown in Fig. 2B. All the enrolled factors were input and studied for potential functional correlations (Fig. S2). Specifically, the main oncogenes ERBB2, KRAS, and TP53 were analyzed for their intrinsic connections in breast carcinomas, and promising correlations between TNFSF4 and ERBB2, KRAS, and TP53 were confirmed (Fig. S3, Table 1).

**Table 1:** The analysis tested in 276 pairs between the 24 tracks in the OncoPrint to reveal the connections among immunotherapies related genes.

A	B	A Not B	B Not A	Both	Log2	P value	q value
TNFSF4	TNFSF18	5	4	70	> 3	< 0.001	< 0.001
TNFSF18	CD48	11	40	63	> 3	< 0.001	< 0.001
TNFSF4	CD48	14	42	61	> 3	< 0.001	< 0.001
CD274	PDCD1LG2	3	1	20	> 3	< 0.001	< 0.001
ERBB2	TP53	369	1345	441	1.409	< 0.001	< 0.001
PVR	NECTIN2	3	9	12	> 3	< 0.001	< 0.001
ERBB2	LGALS9	120	9	27	> 3	< 0.001	< 0.001
TNFSF9	CD70	1	3	7	> 3	< 0.001	< 0.001
HHLA2	CD200	6	3	6	> 3	< 0.001	< 0.001
KRAS	TP53	47	1717	69	1.501	< 0.001	< 0.001
CD80	CD200	2	5	4	> 3	< 0.001	< 0.001
CD86	CD200	7	5	4	> 3	< 0.001	< 0.001
CD274	TP53	10	1013	27	2.355	< 0.001	< 0.001
CD80	CD86	3	8	3	> 3	< 0.001	< 0.001
CD80	HHLA2	3	9	3	> 3	< 0.001	< 0.001
NECTIN2	CD48	12	94	9	2.902	< 0.001	0.001
CD86	HHLA2	8	9	3	> 3	< 0.001	0.003
PVR	CD48	8	96	7	> 3	< 0.001	0.004
CD44	TP53	10	332	19	1.984	< 0.001	0.005
VSIR	TP53	5	338	13	2.421	< 0.001	0.011
CD200	TP53	1	343	8	> 3	< 0.001	0.012
PDCD1LG2	TP53	7	484	14	2.138	< 0.001	0.012
VTCN1	TNFRSF14	27	19	3	> 3	0.001	0.015
CD274	PVR	18	12	3	> 3	0.003	0.031
HHLA2	TP53	3	342	9	2.614	0.004	0.041
ALDH1A1	CD44	10	26	3	> 3	0.004	0.048

The close connections among the candidate immune checkpoints, and the connections among checkpoints and malignancy stimulators of either KRAS, TP53, ERBB2, ALDH1A1, CD44, were all calculated and exhibited. The close connections strongly suggested the crucial roles of candidate immune checkpoints in therapy response, and also suggested the potential effects they may exert.

In general, nearly all the immunotherapeutic targets showed expanded expression intervals in ESR1+ and PGR+ cases, the existence of which demonstrated a better response to standard (“chemotherapeutic”) and anti-hormone treatments, indicating that immunotherapy may compensate for current deficiencies. More importantly and interestingly, TNFSF4, TNFSF18, and CD48 all showed increased expression in breast carcinomas bearing no ERBB2, ESR1, or PGR expression, strongly suggesting that immunotherapy may show efficacy in all types of breast cancer, and a partially enlarged drawing is shown in Fig. 2C. In detail, TNFSF4, TNFSF18 and CD48 showed aberrant expression in breast cancer cases with different genetic backgrounds, including those without KRAS (Fig. 2D), TP53 (Fig. 2E), or ERBB2 (Fig. 2F) expression and activation; these three factors dominated the survival prognosis and led to short predicted survival.

### Evaluation of the cluster of TNFSF4, TNFSF18, CD48 and LGALS9 as therapeutic immune therapeutic targets

**Figure 3:**
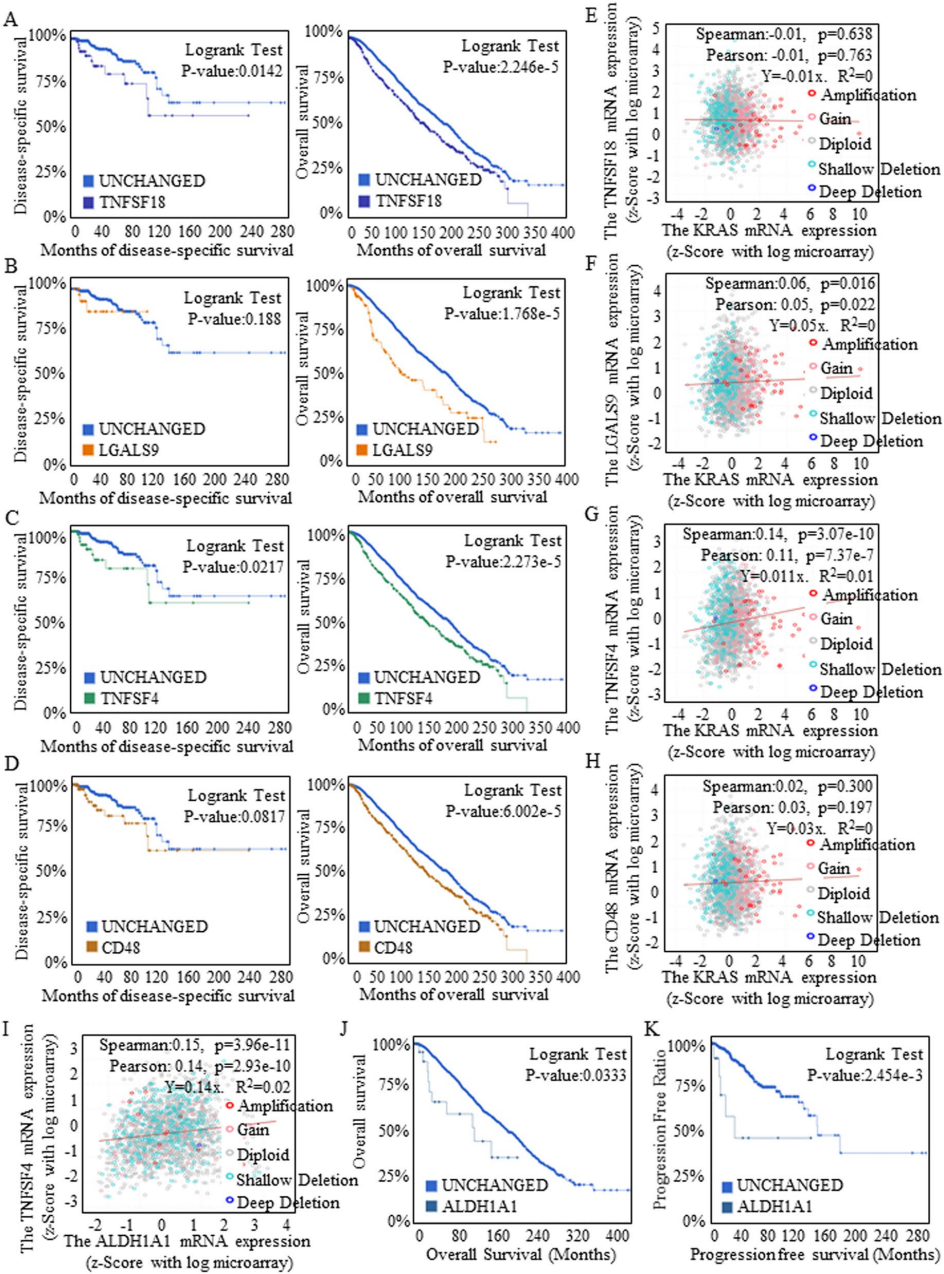
Clinical evaluations of potential therapeutic candidates. Data were acquired from the combined study cohorts of BREAST (METABRIC 2016), BREAST CANCER (MSK 2018), and BREAST INVASIVE CARCINOMA BREAST (TCGA PANCAN 2018). The clinical significance of the cluster including TNFSF18 (**A**), LGALS9 (**B**), TNFSF4 (**C**) and CD48 (**D**) was analyzed for the value of these molecules as immunotherapeutic targets. The correlations between KRAS and TNFSF18 (**E**), LGALS9 (**F**), TNFSF4 (**G**) or CD48 (**H**) were analyzed, and the positive correlation between TNFSF4 and KRAS strongly suggested the prospective therapeutic effects of using TNFSF4 as target for manipulation or blockade. (**I**) A close correlation between TNFSF4 and ALDH1A1 expression was identified. Stem cells with a positive ALDH1A1 phenotype had a shorter survival time (**J**) and shorter progression-free time (**K**).

The clinical significance of a cluster including TNFSF18 (Fig. 3A), LGALS9 (Fig. 3B), TNFSF4 (Fig. 3C) and CD48 (Fig. 3D) was also analyzed for the value of these molecules as immunotherapeutic targets, and TNFSF4 and TNFSF18 showed greater significance in predicting disease-free survival and overall survival (OS). KRAS greatly stimulated carcinogenesis and determined the anticancer treatment response. We further explored the correlations of KRAS with TNFSF18 (Fig. 3E), LGALS9 (Fig. 3F), TNFSF4 (Fig. 3G) and CD48 (Fig. 3H), and the positive correlation between TNFSF4 and KRAS strongly suggested the prospect of achieving therapeutic effects by using TNFSF4 as a target for manipulation or blockade.

Groups of stem cells are considered the root of cancer recurrence due to their steady status and superior renewal ability. We previously identified stem-like ALDH1A1+ cells in breast cancer groups^2,17,18^, and in this study, we noticed a close correlation between TNFSF4 and ALDH1A1 expression (Fig. 3I), the latter of which indicated shorter survival (Fig. 3J) and progression-free times (Fig. 3K). These results strongly suggested that oncogenic TNFSF4 may be an effective immunotherapeutic target, and the proposed survival benefits may also be achieved by repressing stem cell expansion, fully assuring a positive outcome for anti-TNFSF4 treatment. The other candidates, CD112, TNFRSF14, and PD-L1, failed to be involved upon further analysis, as indicated by their negative associations with survival (Fig. S4).

### Probable mechanism by which TNFSF4 functions

**Figure 4:**
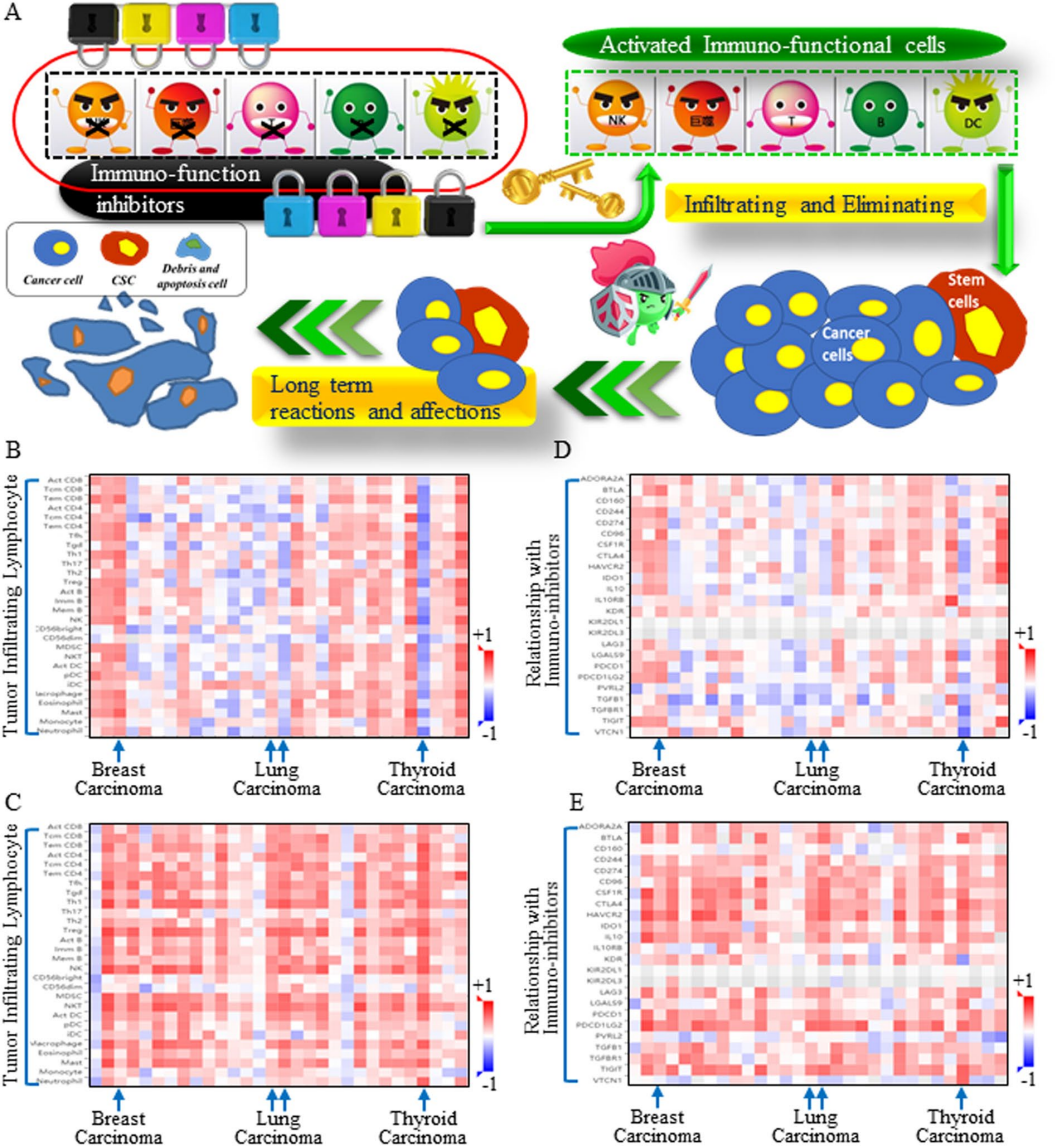
TNFSF4-based immunotherapy may intersect with cancer stem cell signature repression. (**A**) Schematic figure illustrating the immune response. the immune system becomes suppressed when a carcinoma becomes aggressive. Later, when immune function inhibitors are blocked, active immune cells begin to infiltrate and exert cytotoxic activities against all tumor cells. Spearman correlations between TNFSF4 and immunoinhibitory factors (Y axis) across human cancers (X axis). The items in the column are listed in sequence as follows: ADORA2A, BTLA, CD160, CD244, CD274, CD96, CSF1R, CTLA4, HAVCR2, IDO1, IL10, IL10RB, KDR, KIR2DL1, KIR2DL3, LAG3, LGALS9, PDCD1, PDCD1LG2, PVRL2, TGFB1, TGFBR1, TIGIT, and VTCN1. The infiltrating lymphocyte functions and connective functional factors were analyzed, and both ALDH1A1 overexpression (**B**) and TNFSF4 overexpression (**C**) were correlated with more lymphocyte infiltration. Spearman correlations between TNFSF4 and kinds of lymphocytes (Y axis) across human cancers (X axis). The items in the column are listed in sequence as follows: ADORA2A, BTLA, CD160, CD244, CD274, CD96, CSF1R, CTLA4, HAVCR2, IDO1, IL10, IL10RB, KDR, KIR2DL1, KIR2DL3, LAG3, LGALS9, PDCD1, PDCD1LG2, PVRL2, TGFB1, TGFBR1, TIGIT, and VTCN1. However, infiltrating immune cells were suppressed by highly expressed immune inhibitors in cells with increased ALDH1A1 (**D**) or TNFSF4 expression (**E**). These results indicated that TNFSF4 blockade treatment could potentially reactivate the immune response and partially function through precisely inhibiting stem cells.

The immune system becomes suppressed as carcinomas become aggressive, and when immune function inhibitors are applied (immune blockade approaches), active immune cells begin to infiltrate and execute cellular functions (Fig. 4A). All types of cancer cells, including the heterogenetic subtype cells known as cancer stem cells (CSCs), can be eliminated by immune attack. To investigate the crucial role of the selected immune target, infiltrating lymphocyte functions and connective functional factors were analyzed for insights into the underlying mechanisms. Both ALDH1A1 overexpression (Fig. 4B) and TNFSF4 (Fig. 4C) overexpression correlated with more active lymphocytes. However, infiltrating immune cells were suppressed by highly expressed immune inhibitors on cells with increased ALDH1A1 (Fig. 4D) or TNFSF4 expression (Fig. 4E). These results indicated that TNFSF4 can potentially reactivate the immune response and partially function by precisely neutralizing stem cells.

### Stem cell signatures predicted the response to immunotherapy

**Figure 5:**
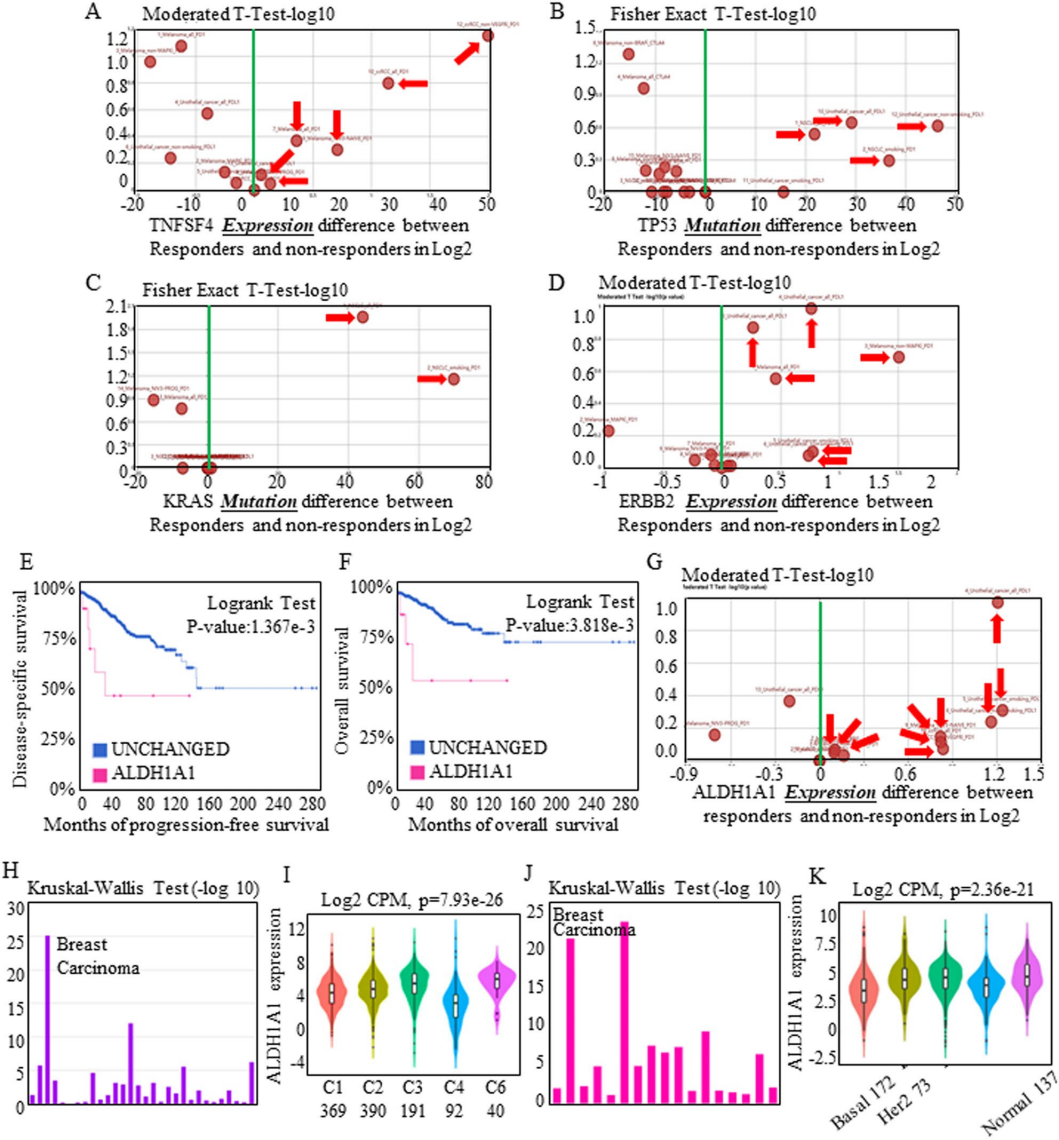
TNFSF4 blockade therapies could be assessed in the stem cell signature-associated mode. The transcriptomic and genomic profiles of pretreated tumor biopsies from responders and non-responders treated with anti-PDL1 and anti-PD1 antibodies were enrolled for analysis. In total, the responders tended to exhibit higher TNFSF4 expression levels, and each study could be reviewed by searching for a certain PMID number (red labeling). Assessment of real-world immunotherapeutic effects indicated that effects related to TNFSF4 (**A**) tended to imply better outcomes and that TNFSF4-associated TP53 (**B**), KRAS (**C**), and ERBB2 (**D**) all indicated better immunotherapy response. Increased ALDH1A1 expression indicated shorter disease-specific survival (**E**) and overall survival (**F**), and ALDH1A1 surprisingly correlated with higher therapeutic response ratios (**G**) through clinical data assessment. (**H**,**I**) ALDH1A1 was analyzed for its roles in predicting immunotherapy response, and the 5 subgroups C1 (N = 369), C2 (N = 390), C3 (N = 191), C4 (N = 92), and C6 (N = 40) were involved in assessing functional aspects. ALDH1A1 expression dominated in all the subtypes, participating in multiple immune reaction processes. (**J**,**K**) Associations between ALDH1A1 expression and molecular subtypes across human cancers were also identified, and the signature of increasing ALDH1A1 expression tended to be found in all kinds of breast carcinomas. Specifically, C1 represents wound healing, C2 represents IFN-gamma dominant, C3 represents inflammatory, C4 represents lymphocyte depleted, C5 represents immunologically quiescent and is not shown, and C6 represents TGF-b dominant.

Immunotherapy responses and effects were believed to be correlated with lymphocyte activation and infiltration, and we first assessed the effects of immunotherapies in multiple systems. Treatments targeting TNFSF4 tended to produce relatively good outcomes (Fig. 5A). Additionally, TNFSF4-associated TP53 (Fig. 5B), KRAS (Fig. 5C), and ERBB2 (Fig. 5D) all indicated a relatively good immunotherapy response, which further supported the crucial position of TNFSF4. Red-labeled text refers to one specific study, and detailed study information can be reviewed by searching certain PMID numbers in TISIDB.

TNFSF4 and ALDH1A1 were confirmed to participate in immune-activating therapies, and TNFSF4 treatment predicted a therapeutic response. Immunotherapy consistently repressed tumor growth once the immune system was activated, and from the above results, we further found that TNFSF4-associated therapy might also influence stem cell expansion. Increased ALDH1A1 expression indicated shorter disease-specific survival (Fig. 5E) and overall survival (Fig. 5F), and in clinical assessments, ALDH1A1 surprisingly correlated with higher therapeutic response ratios (Fig. 5G), which has not ever been reported or analyzed.

Associations between ALDH1A1 expression and immune subtypes across human cancers were included for analysis, and ALDH1A1 expression dominated in all the subtypes (Fig. 5H), participating in multiple immune reaction processes (Fig. 5I). Associations between ALDH1A1 expression and molecular subtypes across human cancers were also identified (Fig. 5J), and a signature of increasing ALDH1A1 levels tended to be observed in all kinds of breast carcinoma (Fig. 5K), indicating the universal therapy-responsive role of ALDH1A1 in achieving better outcomes.

### Preliminary exploration of clinical significance

**Figure 6:**
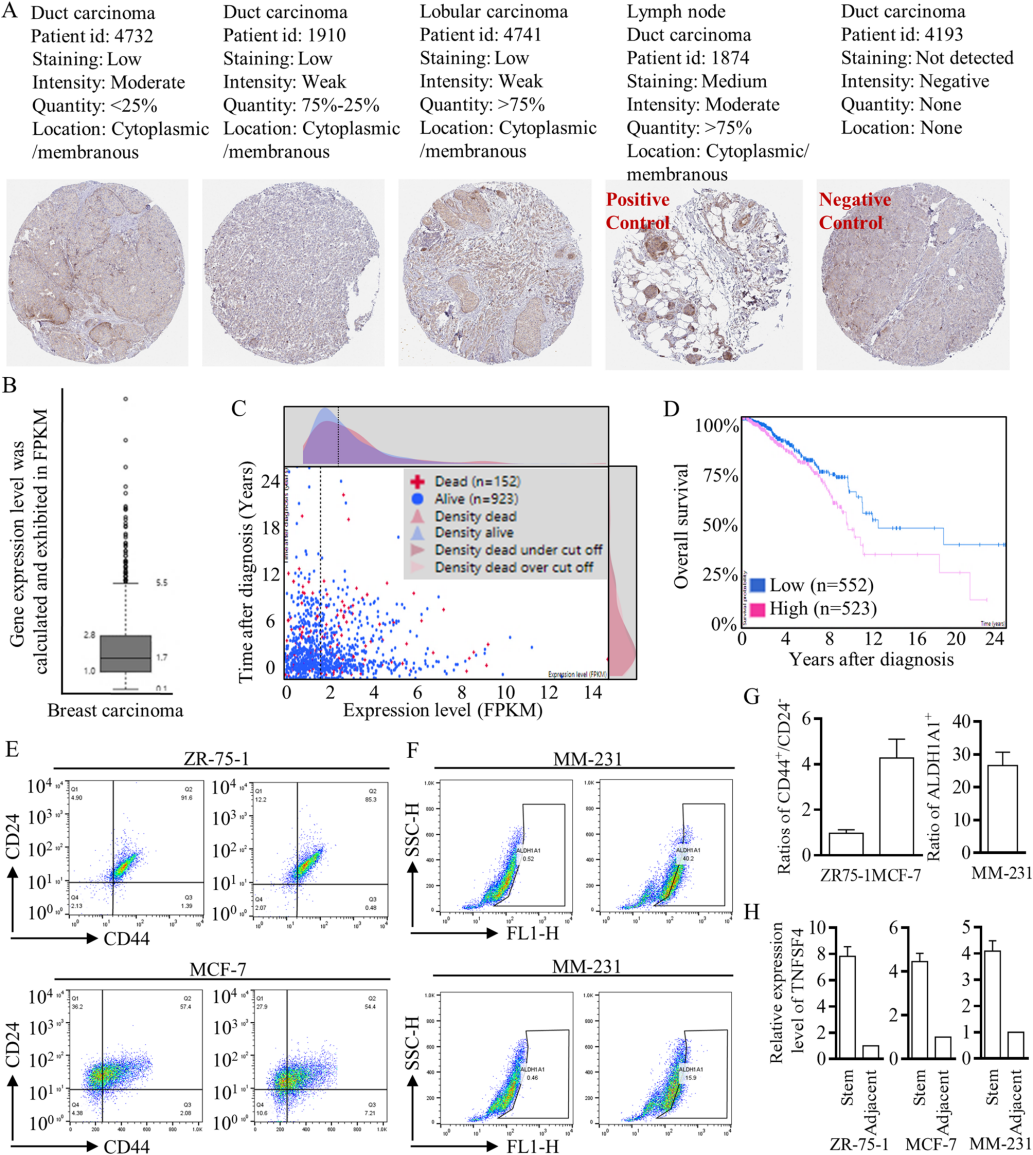
Exploration of the putative clinical roles of TNFSF4. (**A**) IHC staining images are shown to clarify different expression patterns (left to right, in sequence, < 25%, 25–75%, and > 75%). A lymph node slide was set as a positive control, and an unstained slide was set as a negative control. (**B**) High RNA expression of TNFSF4 was universally identified in breast carcinoma, with testing and calculation based on FPKM, and the cutoff line is labeled, which was used for clinical predictions. (**C**,**D**) Higher TNFSF4 expression pointed to poorer survival outcomes. (**E**,**F**) Flow cytometry with FACSAria sorting was applied to isolate stem cells from ZR75-1, MCF-7, and MM-231 cells. (**G**,**H**) Stem cells with a CD44^+^/24^−^ or ALDH1A1^+^ phenotype were identified and isolated, and the TNFSF4 expression patterns in different cell lines were checked to illustrate the increased expression.

Immunohistochemical (IHC) staining images were acquired from “The Human Protein Atlas” project (funded by the Knut & Alice Wallenberg Foundation), and representative images were arranged referring to different groups in the public dataset (Fig. 6A). High expression of TNFSF4 was universally identified in breast carcinoma (Fig. 6B) and pointed to poorer survival outcomes (Fig. 6C,D). To further verify the potential effects of TNFSF4 on stem cell renewal, we isolated stem cells and identified higher TNFSF4 expression in the CD44^+^/24^−^ cell group with the luminal A/B phenotype (Fig. 6E) and in the ALDH1A1^+^ cell group with the basal-like phenotype (Fig. 6F). For the first time, we derived solid results through database screening and bench-to-clinic exploration; the results strongly suggested the value of TNFSF4-based immunotherapy (Fig. 6G,H).

## Discussion

The treatment approaches for breast carcinoma are constantly evolving in accordance with newly confirmed molecular and biological findings, which lead to reconfiguration of treatment methods. Shifting treatment strategies may expand benefits across various carcinomas; however, immunotherapy is not yet effective in most malignancies. Several specific contributions have been identified for breast carcinogenesis, and related therapies that target or relieve the malignant process have greatly improved patient lives. Currently, endocrine therapy, anti-Her-2 therapy, and improved chemotherapy all prolong survival outcomes. We must confront the fact that basal-like breast carcinoma, which is always identified as triple-negative carcinoma, is without any endocrine factors or precise targets for treatment. As an example, immunotherapy has been used across the squamous, adenocarcinoma, and small cell types of lung cancer, indicating universal inhibition. To explore potential effective immune targets, we first input all immunotherapy-related functional factors, and after screening in all kinds of breast carcinomas, several candidates were selected, and their correlations with aggressive breast carcinogens were analyzed to evaluate the possible effects of targeted therapy. To finally identify the most promising immunotherapy candidate, the selected factors were assessed for clinical significance (Fig. 2B).

TNFSF4 belongs to the tumor necrosis factor ligand family and functions in T cell-antigen-presenting cell (APC) interactions, mediating the adhesion of activated T cells to target cells. TNFSF4 can bind to TNFRSF4 and collaboratively stimulate T cell proliferation and cytokine production. There are only limited studies exploring the possible functions of TNFSF4^19,20^, and the roles of TNFSF4 in breast carcinoma are unknown. We found that TNFSF4 was highly expressed in all kinds of breast carcinomas, and its aberrant overexpression was associated with shorter overall survival and disease-free survival. Importantly, TNFSF4 was further revealed to be closely related to antitumor immunity, including multiple immune effector molecules and T cell signatures, as presented and illustrated.

In conclusion, TNFSF4 is a potential immunotherapy target due to its aberrant expression pattern in breast carcinoma, and this protein was even found to be overexpressed in carcinomas without ERBB2, ESR1, or PGR1 amplification and those without either KRAS or TP53 mutation and amplification. TNFSF4, together with other candidates, was included for evaluation, and the positive correlations with immune function inhibitors and lymphocytes drew much attention. In the clinical case analysis, TNFSF4-targeted therapy might show the best response to therapy, and interestingly, TNFSF4 also perturbed stem cell expansion, a signature that is critical for long-term recurrence and response to therapy. Therefore, for the first time, we aimed to identify the key factors that may contribute to breast cancer therapies and provide one potential but crucial approach for treating all types of breast carcinoma with long-term effectiveness.

## Methods and materials

### Database availability and integrated analysis

To analyze immune checkpoint-related prognoses in breast cancer, breast cancer genomics-related datasets in The Cancer Genome Atlas (TCGA, http://cancergenome.nih.gov) and International Cancer Genome Consortium (ICGC, https://icgc.org) were individually collected and subsequently subjected to bioinformatic analysis with the web servers GEPIA2 (http://gepia2.cancer-pku.cn)^21,22^, cBioPortal for Cancer Genomics (http://www.cbioportal.org)^23,24^, and TISIDB (http://cis.hku.hk/TISIDB)^25,26^. In detail, GEPIA2 was used to calculate prognostic indexes, including differential expression, pathological stage, gene correlations, and patient survival; cBioPortal was used to visualize and compare gene alterations; and TISIDB was used to explore correlations between the abundances of immunomodulators and the expression of the investigated genes.

Tumor-infiltrating lymphocytes were analyzed using TISIDB^27^ and the immune-related signatures of 28 tumor-infiltrating lymphocyte types from Charoentong’s study (https://www.cell.com/cell-reports/fulltext/S2211-1247(16)31709-0), which can be viewed on the download page. The relative abundances of tumor-infiltrating lymphocytes were inferred by using gene set variation analysis (GSVA) based on the gene expression profile. Overall survival and disease-free survival analyses were performed using the Kaplan–Meier method with a 50% cutoff to separate the low- and high-expression groups. The log-rank test, also known as the Mantel–Cox test, was used for the hypothesis test. The Cox proportional hazard ratio (HR) and 95% confidence interval were also included in survival plots. A P value < 0.05 was considered statistically significant.

### Clinical data analysis

The cutoff (FPKM) was set based on the FPKM value of each gene. When applying survival curve analysis, patients were classified into two groups, and the association between prognosis (survival) and gene expression (FPKM) was examined. The best expression cutoff referred to the FPKM value that yielded the maximal difference with regard to survival between two groups at the lowest log-rank P value. The best expression cutoff was selected based on survival analysis. Median expression referred to the median FPKM value calculated based on the gene expression (FPKM) data from all patients in the dataset. The median follow-up time referred to the median time (years) after diagnosis with a specific type of cancer based on clinical data from a public dataset.

### Stem cell isolation and RNA quantification

The breast cancer cell lines MCF-7, ZR75-1, and MM-231 were purchased from American Type Culture Collection and maintained in DMEM (Gibco) or RPMI 1640 medium (HyClone). Total mRNA was reverse transcribed into cDNA by using an RT-PCR kit (AT301 TransGen Biotech), and real-time quantitative PCR (RT-qPCR) was performed with a CFX96 Real-Time PCR Detection System (Bio-Rad)^17,18^. For analysis of the expression of the breast cancer stem cell (BCSC) markers CD44 and CD24, cells in different treatment groups were collected and washed with PBS twice. Then, the cells were incubated with anti-CD44-FITC (clone G44-26) and anti-CD24-PE (clone ML5) antibodies (Invitrogen) at 4 °C for 30 min. After two washes, the samples were analyzed using a flow cytometer (FACSCalibur, BD Biosciences)^28,29^. An LSRII flow cytometer (BD Biosciences Pharmingen, San Diego, USA) was used to analyze and separate CSCs based on cell labeling and fluorescence-activated cell sorting. The activated ALDEFLUOR reagent and DEAM purchased from STEMCELL Technologies (Vancouver, BC, Canada) were used to isolate aldehyde dehydrogenase (ALDH+) cells (stem-like cells). The percentages of ALDH1+ stem cells in different groups were analyzed^5,18^.

### Statistical significance and classification

The Spearman method was used to analyze pairwise gene expression correlations, and a P value < 0.05 was considered statistically significant. The correlation degree was identified from the absolute value of the correlation coefficient, and the detailed classifications were as follows: ≤ 0.4, weak; 0.41–0.60, moderate; 0.61–0.80, strong; and 0.81–1.0, very strong. The cooccurrence and mutual exclusivity of genetic alterations between genes of interest and each immune checkpoint molecule were determined from the log2 odds ratio, P value, and Q value. A Q value < 0.05 was considered statistically significant. The investigated immune inhibitors were collected according to Charoentong’s study^27,30,31^, and each Spearman correlation between the investigated genes and a distinct immune inhibitor in an individual cancer type was integrated into the indicated heat-map. Log-rank P values for Kaplan–Meier plots represents results from an analysis of the correlation between an mRNA expression level and patient survival.

### Ethics approval and consent to participate

All procedures performed in the studies involving human participants were in accordance with the ethical standards of the institutional and/or national research committee and with the 1964 Helsinki Declaration and its later amendments or comparable ethical standards for retrospective studies. We confirmed that the IHC results of the human participants were generated in “The Human Protein Atlas” project (www.proteinatlas.org), and the representative images were arranged referring to different groups in the public dataset.

### Consent for publication

The authors declare that each author has approved this article for publication.

## Supplementary Information

Supplementary Information.

## Data Availability

The data and relevant supporting materials related to the findings of this study are available from the corresponding author upon reasonable request.

## Abbreviations

TNFRSF14: TNF receptor superfamily member 14
TNFSF4: TNF superfamily member 4
TNFSF18: TNF superfamily member 18
CD274: PD-L1
DCD1LG2: PD-L2
