# Author Correction: The immunotherapy candidate TNFSF4 may help the induction of a promising immunological response in breast carcinomas

**DOI:** 10.1038/s41598-023-39545-0

**Published:** 2023-08-01

**Authors:** Kai Li, Lei Ma, Ye Sun, Xiang Li, Hong Ren, Shou-Ching Tang, Xin Sun

**Affiliations:** 1 Department of Thoracic Surgery, The First Affiliated Hospital of Xi’an Jiaotong University, 277 Yanta West Road, Xi’an, 710061 Shaanxi China; 2 Department of Thoracic Surgery and Oncology, Cancer Centre, The First Affiliated Hospital of Xi’an Jiaotong University, 277 Yanta West Road, Xi’an, 710061 Shaanxi China; 3 Department of Anesthesiology and Perioperative Medicine, Operating Centre, The First Affiliated Hospital of Xi’an Jiaotong University, Xi’an, 710061 Shaanxi China; 4 Department of Anesthesiology and Operation, Operating Centre, The First Affiliated Hospital of Xi’an Jiaotong University, Xi’an, 710061 Shaanxi China; 5 Department of Pathology, Anatomy and Cell Biology, Sidney Kimmel Cancer Center, Thomas Jefferson University, Philadelphia, PA 19107 USA; 6 University of Mississippi Medical Center, Cancer Center and Research Institute, University of Mississippi, 2500 North State Street, Jackson, MS 39216 USA

Correction to: *Scientific Reports* 10.1038/s41598-021-98131-4, published online 20 September 2021

The original version of this Article contained an error in Figure 6, panel E, where the labeling of the flow cytometry antibody gate and thresholds was incorrect. Additionally, the manually added scale number and the gates data have been deleted. The original Figure 6 and accompanying legend appear below.

**Figure 6:**
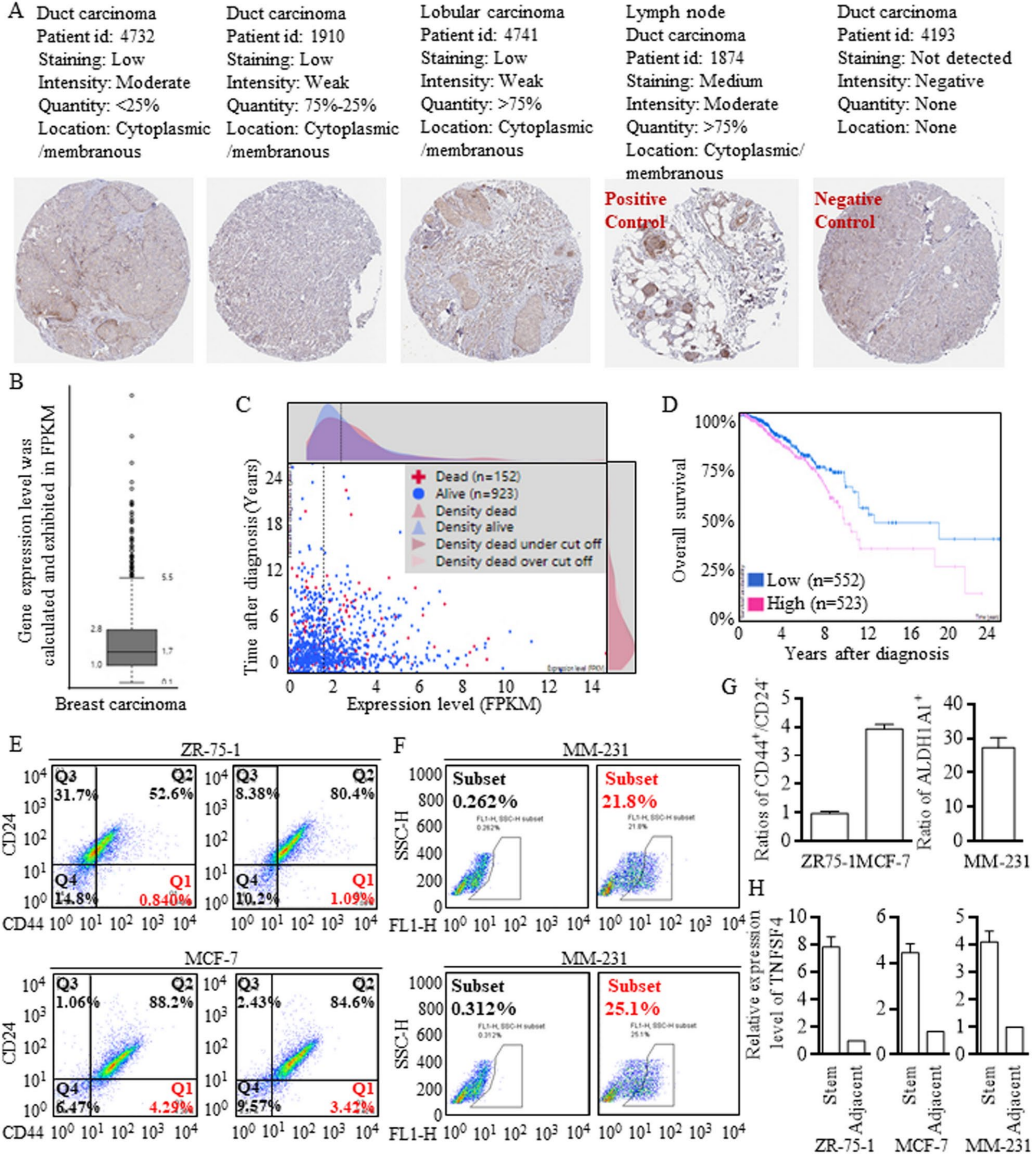
Exploration of the putative clinical roles of TNFSF4. (**A**) IHC staining images are shown to clarify different expression patterns (left to right, in sequence, < 25%, 25–75%, and > 75%). A lymph node slide was set as a positive control, and an unstained slide was set as a negative control. (**B**) High RNA expression of TNFSF4 was universally identified in breast carcinoma, with testing and calculation based on FPKM, and the cutoff line is labeled, which was used for clinical predictions. (**C**,**D**) Higher TNFSF4 expression pointed to poorer survival outcomes. (**E**,**F**) Flow cytometry with FACSAria sorting was applied to isolate stem cells from ZR75-1, MCF-7, and MM-231 cells. (**G**,**H**) Stem cells with a CD44^+^/24^−^ or ALDH1A1^+^ phenotype were identified and isolated, and the TNFSF4 expression patterns in different cell lines were checked to illustrate the increased expression.

The original Article has been corrected.

