# Supplementary material for: The immunotherapy candidate TNFSF4 may help the induction of a promising immunological response in breast carcinomas
Source: Sci Rep. 2021 Sep 20;11:18587. doi: 10.1038/s41598-021-98131-4 (PMC8452722; doi:10.1038/s41598-021-98131-4)

**Supplemental Figure Legends**

**Supplemental Figure Legend 1**

The body-maps illustrating the representative immunity therapeutics were drafted to visualize the distributions, extents, and intentions in multiple systems. The red depicts and the green depicts indicated the overexpression and the repression respectively.


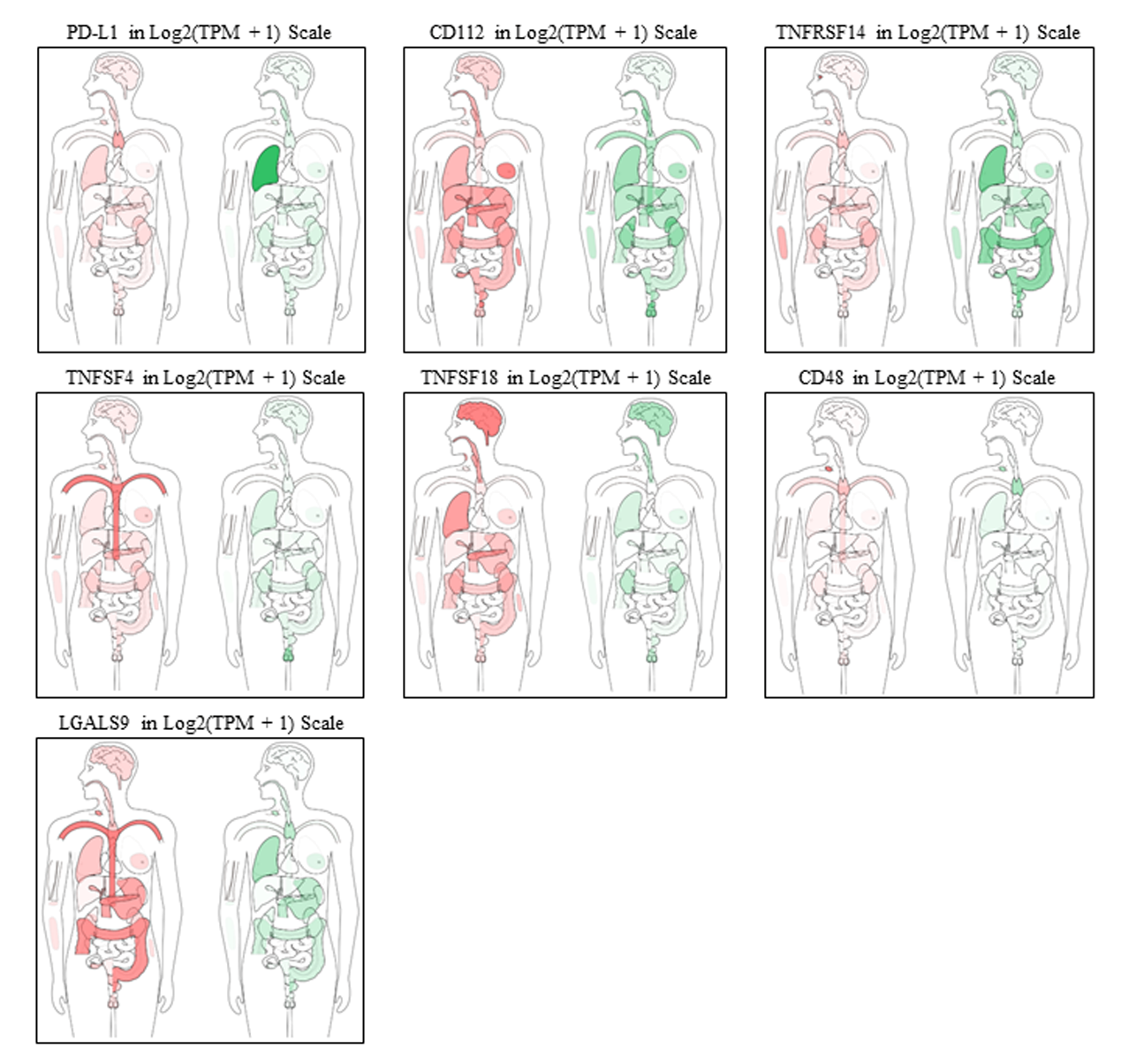


**Supplemental Figure Legend 2**

The potential immunity therapeutics of ADORA2A, BTLA, Nectin-2 (CD112), CD160, CD244, PD-L1 (CD274), CD96, CSF1R, CTLA4, HAVCR2, IDO1, IL10, IL10RB, KDR, KIR2DL1, KIR2DL3, LAG3, LGALS9, PDCD1, PDCD1LG2, PVRL2, TGFB1, TGFBR1, TIGIT, VTCN1, TNF Receptor Superfamily Member 14 (TNFRSF14), TNF superfamily member 4 (TNFSF4), TNF superfamily member 18 (TNFSF18), were all input for studied for potential functional correlations, and the illustration was drafted through using GEPIA2 online analysis.


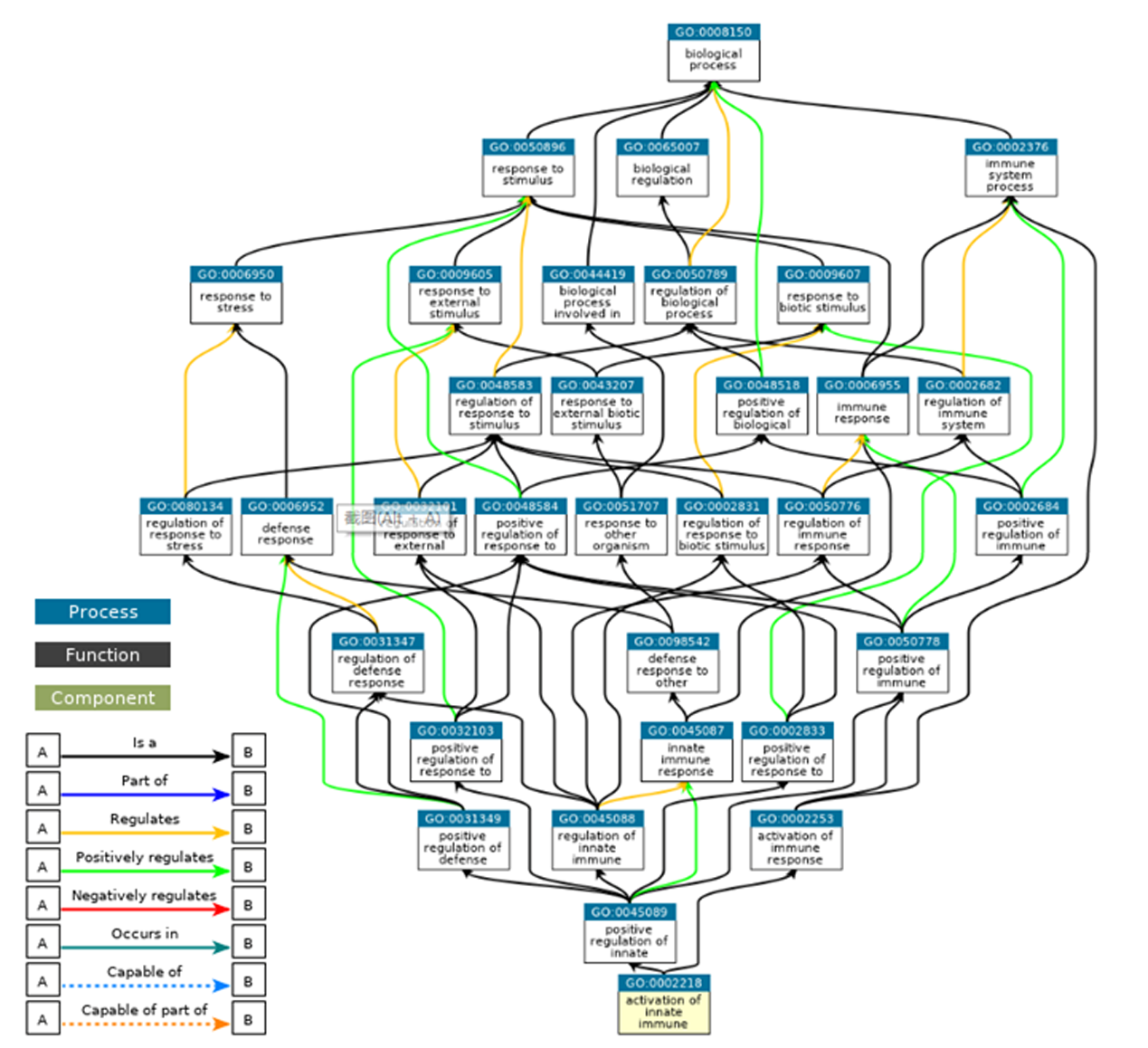


**Supplemental Figure Legend 3**

The main carcinogens of ERBB2, KRAS, TP53 were analyze for their intrinsic connection. Among the candidates, only TNFSF4 was positively correlated with KRAS and TP53.


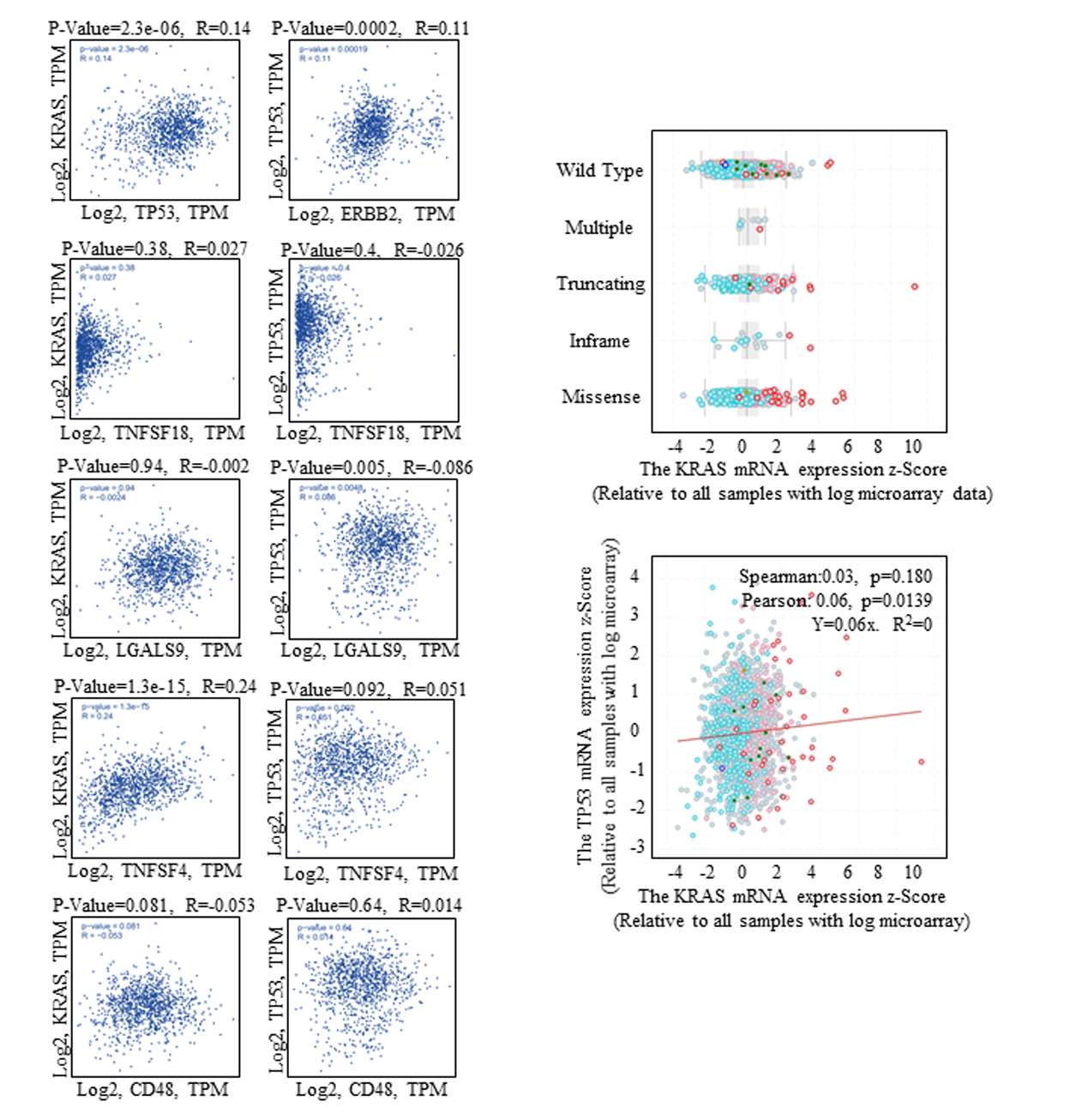


**Supplemental Figure Legend 4**

The therapeutic candidates of CD112, TNFRSF14 and PD-L1 were analyzed for potential clinical significances, and they failed to be involved in further analysis, as their negative indicating roles in either disease-specific survival or overall survival.


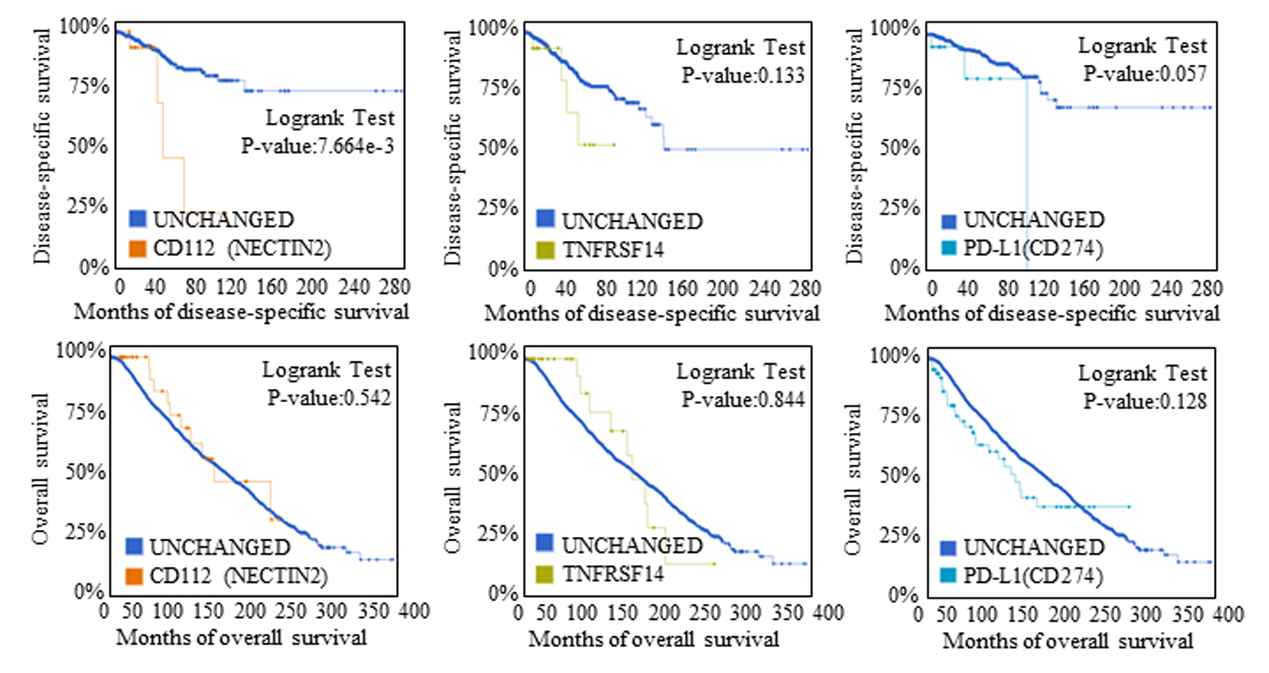

Supplement: Supplementary file 1 — Supplementary Information. [file 41598_2021_98131_MOESM1_ESM.docx]
